# Genetic Characterization of Some Saudi Arabia’s Accessions from *Commiphora gileadensis* Using Physio-Biochemical Parameters, Molecular Markers, DNA Barcoding Analysis and Relative Gene Expression

**DOI:** 10.3390/genes13112099

**Published:** 2022-11-11

**Authors:** Fatmah Ahmed Safhi, Salha Mesfer ALshamrani, Areej Saud Jalal, Diaa Abd El-Moneim, Amal A. Alyamani, Amira A. Ibrahim

**Affiliations:** 1 Department of Biology, College of Science, Princess Nourah bint Abdulrahman University, P.O. Box 84428, Riyadh 11671, Saudi Arabia; 2 Department of Biology, College of Science, University of Jeddah, Jeddah 21959, Saudi Arabia; 3 Department of Plant Production(Genetic Branch), Faculty of Environmental and Agricultural Sciences, Arish University, El-Arish 45511, Egypt; 4 Department of Biotechnology, Faculty of Science, Taif University, Taif 21974, Saudi Arabia; 5 Botany and Microbiology Department, Faculty of Science, Arish University, El-Arish 45511, Egypt

**Keywords:** *Commiphora gileadensis* L., SCoT, DNA barcoding, genetic diversity, gene expression, ISSR, physio-biochemical parameters

## Abstract

*Commiphora gileadensis* L. is a medicinal plant, known as balsam, with pharmaceutical potential for its phytochemical activities and chemical constituents. Genetic diversity is a genetic tool used in medicinal plant evolution and conservation. Three accessions from *C. gileadensis* were collected from three localities in Saudi Arabia (Jeddah, Jizan and Riyadh). Genetic characterization was carried out using physio-biochemical parameters, molecular markers (inter-simple sequence repeat (ISSR) and start codon targeted (SCoT)), DNA barcoding (18 S rRNA and ITS rDNA regions), relative gene expressions (phenylalanine ammonia-lyase 1 (PAL1), defensin (PR-12)) and pathogenesis-related protein (AFPRT). The results of this study showed that *C. gileadensis* accession C3, collected from Riyadh, had the highest content from the physio-biochemical parameters perspective, with values of 92.54 mg/g and 77.13 mg/g for total phenolic content (TPC) and total flavonoid content (TFC), respectively. Furthermore, the highest content of antioxidant enzyme activity was present in accession C3 with values of 16.87, 60.87, 35.76 and 27.98 U mg^−1^ for superoxide dismutase (SOD), peroxidase (POD), catalase (CAT) (mol/min/mg FW) and ascorbate peroxidase (APX) (U mg^−1^ protein), respectively. The highest total number of bands and number of unique bands were 138 and 59, respectively, for the SCoT marker. The SCoT marker was the most efficient for the genetic diversity of *C. gileadensis* by producing the highest polymorphism (75.63%). DNA barcoding using 18 S and ITS showed the nearby *Commiphora* genus and clustered *C*. *gileadensis* accessions from Jeddah and Jizan in one clade and the *C. gileadensis* accession from Ryiadh in a separate cluster. Moreover, relative gene expression of the PAL1, defensin (PR-12) and AFPRT (PR1) genes was upregulated in the *C*. *gileadensis* accession from Ryiadh. In conclusion, ecological and environmental conditions in each locality affect the genomic expression and genetic diversity, which can help the evolution of important medicinal plants and improve breeding and conservation systems.

## 1. Introduction

Genus *Commiphora* Jacq., which belongs to the Burseraceae family, contains about 185 species that are found in tropical and subtropical areas [1,2]. Species in the genus *Commiphora* generate fragrant resins that are used to make incense, perfume and medicines for rheumatism, jaundice, scurvy and liver ailments [3,4,5]. This genus is characterized by small trees with thorny, short branches. The *Commiphora* genus is distributed along the Red Sea shore in western and southwest Saudi Arabia [6,7]. Six species from genus *Commiphora* are present in Saudi Arabia as follows: *C. erythraea*, *C. gileadensis*, *C. habessinica*, *C. kataf*, *C. myrrha* and *C. quadricincta* [8,9]. The life history of the genus *Commiphora*, which includes a deciduous habit, a largely dioecious breeding system, and a tendency to produce flowers before producing leaves, has hampered a systematic understanding at the species level [10]. One of the *Commiphora* species is *C. gileadensis* L., also known as balsam and commonly known as the balm of Gilead is used in folk medicine in the Arabian region due to its bioactive constituents such as phenols, flavonoids and alkaloids. 

DNA barcoding is a novel strategy based on the variety of nucleotide sequences seen in different animals as a result of the development of molecular genetics. A traditional method for identifying medicinal plants is based on their morphology; however, DNA-based molecular approaches are increasingly being used for taxonomic identification [11]. The internal transcribed spacer (ITS) sequence of nuclear ribosomal DNA, which is easier to amplify by universal primer, shorter in length and capable of greater species-level discrimination, is a smart gene for producing species-specific phylogenetic conclusions in most plant groups [12,13]. DNA barcoding is quickly developing to encompass genome skimming. The second internal transcribed spacer (ITS) was recommended as a universal DNA barcode, and that ITS/ITS2 should be integrated as a fundamental barcode for seed plants [14,15,16].

An important first step in creating plant breeding programs is a genetic diversity study. To develop plans for breeding, conserving and using the genetic resources of a crop’s wild relatives, particularly those with a main gene pool, it is imperative to have a deeper understanding of the genetic relationships across plant species’ germplasms, both cultivated and wild [17]. DNA molecular markers, biochemical protein analysis (SDS-PAGE, isozyme test), phytochemical and agronomic evaluation, as well as other approaches, are generally employed to measure genetic diversity [18].

Variations in secondary metabolites include phytochemical markers for species identification. The genetic make-up of the genotype and environmental factors both contribute to phytochemical variation in wild plant species. A difference in chemotypes has been observed based on a variety of environmental factors such as vegetation type, topographical parameters, climatic conditions and pedological features [19,20]. Phyto-biochemical diversity is a desired tool utilized in conventional and molecular breeding programs [21]. 

Changes in environmental conditions, natural habitats as well as deforestation, overgrazing and urbanization affect the genetic diversity of plants. Molecular markers have aided in resolving this problem by clarifying data relevant to diversity, which is helpful for formulating various conservative strategies [22]. SDS-PAGE for storage protein profiling can be used for a variety of tasks, including characterization of species identification, biosystematics analysis, determining the phylogenetic relationship between different species and the generation of pertinent information to support evaluation [23,24,25]. DNA markers such as random amplified polymorphic DNAs (RAPD) and inter simple sequence repeat (ISSR) markers have proven to be highly helpful tools for quickly assessing genetic differences across accessions, especially when genome sequence information is absent [26].

In plants, a potent gene-targeting marker system called start codon targeted polymorphism (SCoT) is dependent on conserved areas that surround the start codons (ATG) of genes. It is extremely reproducible and has a high potential for genetic resolution; however, it is reliant on primers with relatively high annealing temperatures [27,28]. Another effective technique that uses all experimental conditions to construct a co-expression network is gene expression. Its simplicity makes it a potent tool for finding transcriptional modules compared to a bi-clustering analysis [29]. Many studies have used molecular and genetic methods to silence the PAL gene to study its biological functions in plant growth, development and environmental stress [30]. A promising method for demonstrating the plant environment response mechanism uses methodologies that combine DNA marker and gene expression techniques. 

The main aim of the present investigation is to study the genetic diversity between three accessions of *C. gileadensis* in Saudi Arabia based on different tools: (a) phyto/biochemical analyses; (b) DNA barcoding (18 S rRNA and ITS rDNA); (c) molecular markers (ISSR and SCoT); (d) relative gene expressions for some genes (PAL1, defensin, (PR12) and AFPRT (PR1)). 

## 2. Materials and Methods

### 2.1. Plant Material 

Three accessions of *C. gileadensis* collected from three localities in KSA and the code of the studied accession are presented in Table S1.

### 2.2. Phytochemical and Enzyme Antioxidant Activities

#### 2.2.1. Phytochemical Parameters

About 5 mg of leaf powder that had been air dried was added to 10 mL of methanol, and using the Folin–Ciocalteu reagent technique, the total phenolic content (TPC) for all accessions was calculated [31]. The aluminum chloride colorimetry method was used to determine the total flavonoid content (TFC) for all accessions [32].

For tannins, Makkar [33] demonstrated the use of polyvinyl-pyrrolidone (PVP) to bind tannins for the measurement of total tannins (TTs). In a glass test tube, a 100 mg sample of polyvinyl pyrrolidone was weighed, 1.0 mL of distilled water and 1.0 mL of the extract with tannins were then added, the test tube was then kept at 4 °C for approximately 10–15 min. It was then centrifuged for 10 min at 3000 rpm. The supernatant was then collected using a straightforward phenolic compound that did not include tannins, and the absorbance or UV reading was taken, as previously indicated, and expressed as tannin acid equivalent (TAE). The difference between the amount of simple phenols and total phenol (TP) in the extract was used to calculate the total tannin content.

The Hiai et al. approach, with a few minor adjustments, was used to test the total saponins content (TSC) colorimetrically [34]. This method is based on the production of chromophores that absorb light at 544 nm by the interaction of vanillin and sulfuric acid with the C-3 carbon of saponins. About 0.5 mL of the fresh extracts was collected, and they were rotavaporized until dry. The residue was dissolved in 10 mL of an aqueous solution of 80% methyl alcohol. The methanolic solution was combined with 5 mL of 72% sulfuric acid solution, 0.5 mL of 8% vanillin solution in ethanol, and 0.5 mL of the methanolic solution. The vial of this concoction vial was chilled in a water-ice mixture for four minutes after being submerged in a 60 °C water bath for ten minutes. Using a Shimadzu UV mini-1240 UV/vis Scanning Spectrophotometer, 115 VAC, the absorbance was measured at 544 nm (Duisburg, Germany). Using a standard curve corresponding to a solution containing 40–550 mg/L of diosgenin, the results were expressed as milligrams of diosgenin equivalents per 1 g of dry matter (mg DE/g DM).

#### 2.2.2. Enzyme Antioxidant Activity

By utilizing a prechilled pestle and mortar to homogenize 1 g of fresh *C. gileadensis* leaf tissue in cooled 50 mM phosphate buffer (pH 7.0), 1% polyvinyl pyrrolidine, and 1 mM EDTA, antioxidant enzymes were extracted. The supernatant was used for enzyme testing after centrifuging at 18,000× *g* for 30 min at 4 °C. In a 1.5 mL assay mixture containing sodium phosphate buffer (50 mM, pH 7.5), 100 L EDTA, L-methionine, 75 M NBT, riboflavin, and 100 L enzyme extract, the activity of superoxide dismutase (SOD, EC 1.15.1.1) and NBT photochemical reductions were observed at 560 nm. The light was turned off after 15 min of incubation, and the activity was expressed as EU mg^−1^ protein. The catalase activity assay (CAT, EC1.11.1.6) was carried out in accordance with the method described by Luck [35]. The change in absorbance was observed at 240 nm for 2 min, and the calculation employed the extinction coefficient of 39.4 mM^−1^ cm^−1^. By watching the change in absorption at 290 nm for three minutes while a reaction mixture containing potassium phosphate buffer (pH 7.0), 0.5 mM ascorbic acid, hydrogen peroxide and enzyme extract was added to a 1 mL test tube, the ascorbate peroxidase activity assay (APX, EC 1.11.1.11) was performed. An extinction coefficient of 2.8 mM^−1^ cm^−1^ was calculated. Peroxidase (POD, EC, 1.11.1.7) activity was assayed according to Zhou and Leul [36]. 

### 2.3. SDS-PAGE Protein

Using about 20 mM Tris-Cl extraction buffer (pH 8.0) containing two mM EDTA, one mM PMSF, the proteins from the leaves of three *C. gileadensis* were isolated. Each sample’s protein content was calculated using Bradford’s formula [37]. The extracted protein from the leaves underwent SDS-PAGE (sodium dodecyl sulphate-polyacrylamide gel electrophoresis) on 15% polyacrylamide gel in accordance with Laemmli’s technique [38]. Each accession’s electrophoretic profile of the proteins found in the leaves was quantified as either (1) the presence or (0) the absence of a band with a certain molecular weight.

The Bio-Rad Gel Documentation System (BIO-RAD-Gel-Doc Model 2000) was used to assess the protein profile. The experiment was carried out in the Plant Laboratory, Faculty of Science, Arish University, Egypt (August 2022).

### 2.4. Molecular Analysis

Molecular analysis using the CTAB buffer methodology outlined by Cota-Sanchez et al. [39], was carried out at the Plant Laboratory, Faculty of Science, Arish University, Egypt (August 2022). The young leaves of three accessions from *C. gileadensis* were used for DNA extraction, and the concentration was measured using nanodrop. In this investigation, ten primer pairs from the ISSR and eleven primers from the SCoT marker were employed. Table 3 provides information on the primers’ names and sequences. According to Zietkiewicz et al. [40] and Collard and Mackill [41], DNA amplification was carried out in 20 μL of a PCR reaction mixture that contained 10 μL of master (2X TOPsimpleTM DyeMIX-nTaq), 5 µL of (0.1µM) for each primer and 1 μL of genomic DNA (50 ng/ μL). A final volume of 20 μL was achieved using sterile distilled water. A PCR reaction condition using The SimpliAmp™ Thermal Cycler was as follows: predenaturing for 5 min at 95 °C, then 45 cycles at 94 °C for 30 s, annealing for 40 s, and 72 °C for 1 min, with a final incubation duration of 5 min at 72 °C. On a 1.5% agarose gel, the products were separated. For each accession, the data were scored as (1) for presence and (0) for absence. Several parameters were computed to evaluate the markers’ informativeness in separating the investigated accessions. The formula for determining polymorphism information content (PIC) is PIC = 1 − Σpi^2^, where pi is the frequency of the ith allele [42]. The effective multiplex ratio (EMR): EMR is equal to np (np/n), where np is the total number of polymorphic loci and n is the number of polymorphic loci (per primer). The marker index (MI) was conducted using the formula MI = PIC x EMR [43,44]. The resolving power (RP) of each primer was calculated using the formula RP = Σ Ib, where Ib is band informativeness (the Ib can be represented on a scale of 0–1 by the following formula: Ib = 1 − (2 − (0.5 − p)), where p is the proportion of accessions containing the band [45]. 

### 2.5. DNA Barcoding 

The DNeasy 96 Plant Mini Kit was used to extract DNA from about 0.5 g of three studied accessions (QIAGEN, Hilden, Germany). Using primers ITS1 (5′-GGAAGTAAAAGTCGTAACAAGG-3′) and ITS4 (5′-ATCCTCCGCTTATTGATATGC-3′) for the ITS region and 18 S primer for the rRNA region was (5′-AACCTGGTTGATCCTGCCAGT-3 F) and (5′-GGCACCAGACTTGCCCTC-3′) for PCR amplification to identify DNA barcoding for the studied accessions. The purified DNA underwent DNA sequencing after being purified using a QIAquick PCR purification kit from QIAGEN, Hilden, Germany. Big DyeTM Terminator Cycle Sequencing Kits were used in an automatic sequencer ABI PRISM 3730XL analyser to perform the product PCR’s sequencing (Microgen Company, Moscow, Russia) (http://www.ncbi.nlm.nih.gov/BLAST, accessed on 14 August 2022). The phylogenetic tree was created using MEGA 10 software and the UPGMA (Unweighted Pair Group Method with Arithmetic Mean) statistical approach (https://www.megasoftware.net/, accessed on 14 August 2022).

### 2.6. Gene Expression

#### 2.6.1. RNA Extraction and cDNA Synthesis

The relative gene expressions of the studied genes (PAL1, defensin (PR-12) and AFPRT (PR1)) were quantitatively measured in the three *C. gileadensis* accessions’ leaves. Total RNA was isolated from 0.5 g sample by using the TRIZOL reagent (Invitrogen, Waltham, MA, USA) according to the manufacturer’s protocol. The purified RNA was analyzed on 1% agarose gel. For each sample, to obtain cDNA, 10 µL total RNA was treated with DNAse RNAse-free (Fermentas, Waltham, MA, USA), 5 μL of which was reverse transcribed in a reaction mixture consisting of oligo dT primer (10 pmL/μL), 2.5 μL 5X buffer, 2.5 μL MgCl2, 2.5 μL 2.5 mM dNTPs, 4 μL from oligo (dT), 0.2 μL (5 Unit/μL) reverse transcriptase (Promega, Walldorf, Germany) and 2.5 μL RNA. The cDNA amplification was performed in a thermal cycler PCR at 42 °C for 1 h and 80 °C for 15 min.

#### 2.6.2. Real-Time Quantitative PCR Analysis

The quantitative study was carried out to evaluate the temporal expression of studied genes in all accessions. Quantitative real-time PCR was carried out on 1 μL diluted cDNA by triplicate using a real time analysis system (Rotor-Gene 6000, Qiagen, Hilden, Germany). Primers of three genes and a housekeeping gene (reference gene), as listed in Table S2, were used for gene expression. The analysis used a SYBR^®^ Green based method; a total reaction volume of 20 μL was used. The reaction mixture consisted of 2 μL of template, 10 μL of SYBR Green Master Mix, 2 μL of reverse primer, 2 μL of forward primer, and sterile distilled water for a total volume of 20 μL. PCR assays were performed using the following conditions: 95 °C for 15 min followed by 40 cycles of 95 °C for 30s and 58 °C for 30 s. The CT of each sample was used to calculate ΔCT values (target gene CT subtracted from β-Actin gene CT). The relative gene expression was determined using the method 2^−ΔΔCT^ [46]. A real time experiment was conducted in the Plant Laboratory, Faculty of Science, Arish University, Egypt (August 2022).

### 2.7. Statistical Analysis 

All data are represented as the mean ± SD of three replicates. The statistical analysis was carried out using the statistical package SPSS for social sciences (SPSS) 16. All outfindings were analyzed using one-way ANOVA (analysis of variance), performed for significance difference at the *p* < 0.05 level; comparisons among different accessions were performed using Duncan’s multiple range tests. Moreover, the heatmap and color-matrix correlation were created to compare and contrast the studied phyto-biochemical parameters and antioxidant enzyme activity using R language. PAST was used to calculate principal component analysis (PCA) and a UPGMA cluster dendrogram of the investigated accessions (ver. 4, Past Software University of Oslo, Oslo, Norway). 

## 3. Results

### 3.1. DNA Barcoding 

For the ITS region, the sequence length and GC content for the three accessions are illustrated in Table 1, which varied from 420 bp for C2 to 1517 bp for C1 and 56% for C1 to 64.3% for C3, respectively. The region of ITS (rDNA) used as the identity in the BLAST search (https://blast.ncbi.nlm.nih.gov/Blast.cgi, accessed on 14 August 2022) showed the similarity of C1 with C2, having a pairwise identity (PI) of 100%; the PI was 88.57% for *C. myrrha* (KC311151), and C3 showed a 95.45% PI for *C. gileadensis* (MH522402).

For 18 S rRNA, the sequence length and GC content for the studied accessions ranged from 753 bp in C3 to 859 bp in C2 and 50.1% for C1 to 62.7% in C2. The BLAST search (https://blast.ncbi.nlm.nih.gov/Blast.cgi, accessed on 14 August 2022) showed a pairwise identity (PI) of 100% for C1 with C3 and 98.39% with *Schinus molle* (AF207015). The pairwise identity for C2 was 91.13% with *Commiphora* sp. 18 S (MN257655). The nuclear DNA result from the ITS and 18 S rRNA regions showed the usefulness of these genes for barcoding due to the phylogeographic variations.

**Figure 1:**
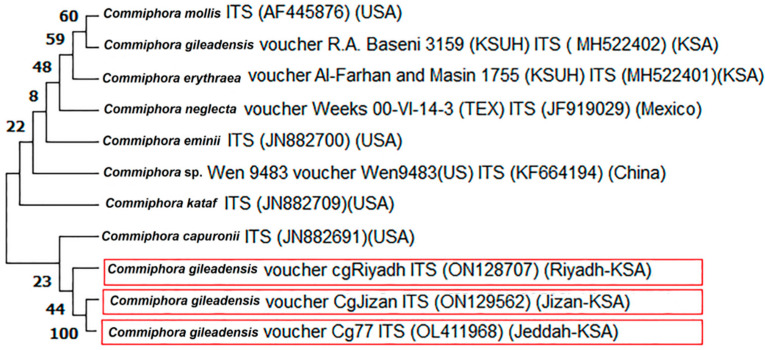
Phylogeny tree for *C. gileadensis* accessions in KSA from ITS sequences (highlighted in red) compared with other relatives of the closest species obtained from GenBank from different localities. Bootstrap tests were performed with 2000 replications.

**Figure 2:**
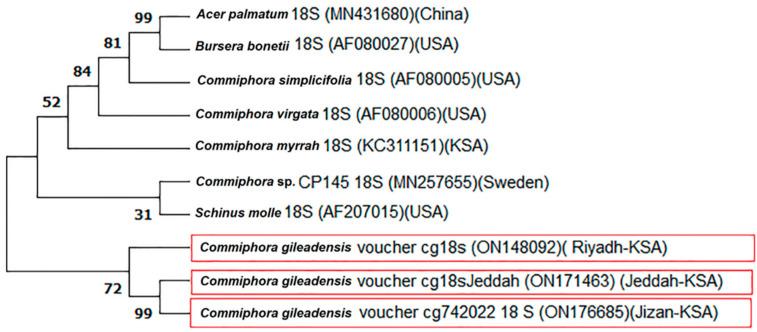
Phylogeny tree for *C. gileadensis* accessions in KSA from 18 S rRNA sequences (highlighted in red) compared with other relatives of the closest species obtained from GenBank from different localities. Bootstrap tests were performed with 2000 replications.

The phylogenetic analysis for the ITS gene is presented in Figure 1. The phylogenetic tree showed two main clades; the large clade included different *Commiphora* species from different countries. The small clade included the studied accessions of *C. gileadensis* with *C. capuronii* from the USA. The first sub-clade of the divided small clade, included *C. capuronii* with bootstrap support (23% BS). The accessions were classified into two sub-clades; the first sub-clade contained C1 and C2 with bootstrap support (100% BS) and the second sub-clade contained the accession C3 (44% BS). A molecular phylogenetic tree for the 18 S rRNA gene was created (Figure 2) and showed a tree classified into two main clades. The large clade included four *Commiphora* species and *Acer palmatum*, *Bursera bonetii* and *Schinus ole*. The three accessions were classified into the second small clade, which was also classified into two sub-clades. The first sub-clade contained C1 and C2 with bootstrap support (99% BS) and the second sub-clade contained C3 with bootstrap support (72% BS).

### 3.2. Phyto-Biochemical and Enzyme Activity Analysis

**Figure 3:**
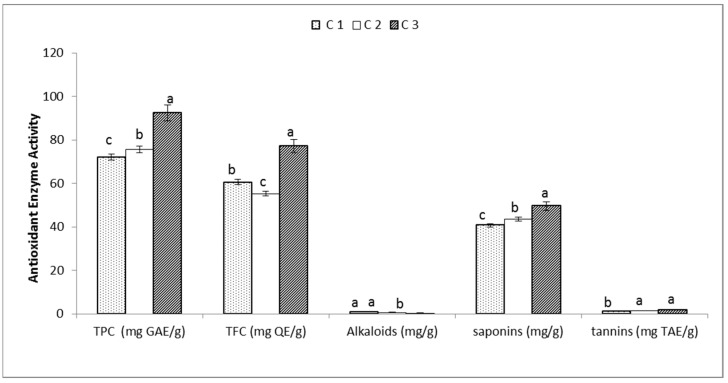
Phytochemical parameters for the studied *C. gileadensis* accessions (C1: collected from Jeddah, C2: collected from Jizan and C3: collected from Riyadh); TPC: total phenolic content; TFC: total flavonoids content. Bars with different letters indicate significant differences between treatments at *p* ≤ 0.05. Data are expressed as the mean of three replicates ± SDs.

**Figure 4:**
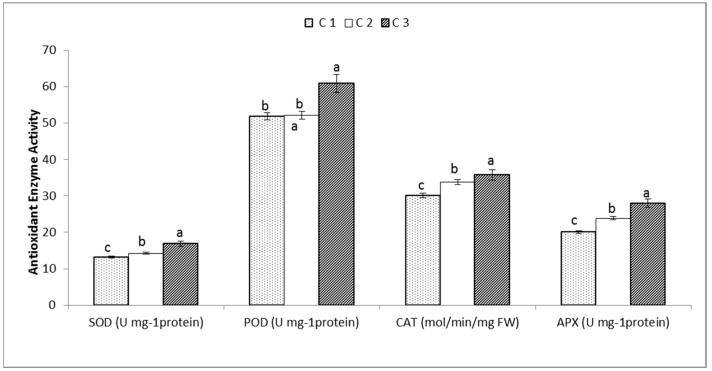
Antioxidant enzyme activity for the studied *C. gileadensis* accessions (C1: collected from Jeddah, C2: collected from Jizan and C3: collected from Riyadh). Bars with different letters indicate significant differences between accessions, expressed as the mean of three replicates ± SDs.

**Figure 5:**
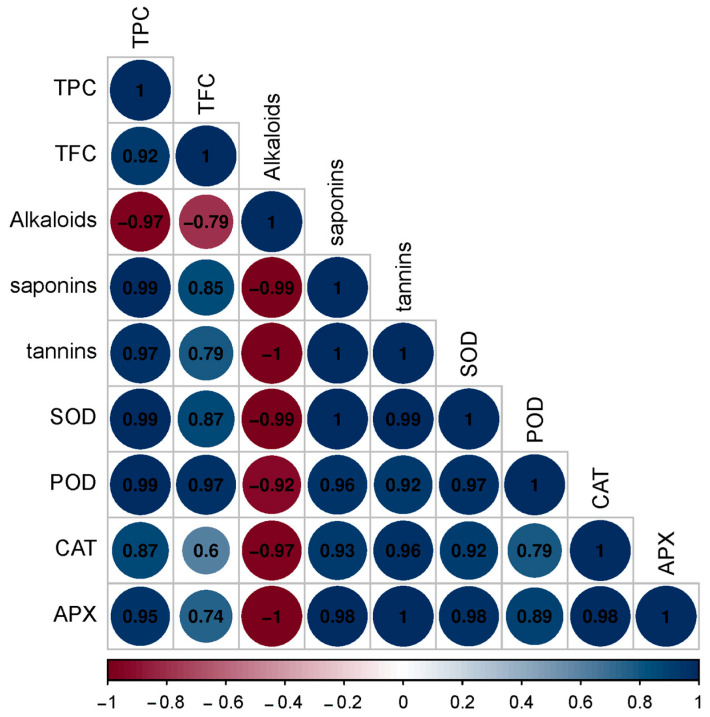
Color plot correlation among the phytochemical parameters and enzyme activity for *C. gileadensis* accessions. The size of circle represents the strength of the correlation, greater the circle stronger the association; TPC: total phenolic content; TFC: total flavonoids content; SOD: superoxide dismutase; POD: peroxidase; CAT: catalase; APX: peroxidase.

The phytochemical analysis was illustrated by the estimation of the total phenolic content (TPC), total flavonoid content (TFC), alkaloids, saponins and tannins as shown in Figure 3. The highest TPC and TFC were present in C3 with a value of 92.54 (mg GAE/g) and 77.13 (mg QE/g), respectively. The highest content from alkaloids was 0.87 (mg/g) in C1; the highest amount of saponins and tannins were found in C3 with a value of 49.67 (mg/g) and 1.87 (mg TAE/g), respectively, followed by C2 with a value of 43.65 (mg/g) to 1.43 (mg TAE/g). Enzyme antioxidant activities for superoxide dismutase (SOD), peroxidase (POD), catalase (CAT) and ascorbate peroxidase (APX) were estimated for the three accessions of *C. gileadensis* as shown in Figure 3. The data showed that the accession C3 collected from Riyadh had the highest content of SOD, POD, CAT and APX with 16.87 U mg^−1^ protein, 60.87 Umg^−1^ protein, 35.76 mol/min/mg FW and 27.98 Umg^−1^ protein, respectively (Figure 4). A Pearson correlation matrix of the phyto-biochemical and enzyme activity parameters is shown in Figure 5. The highest positive correlation was 1.00 between saponins and tannins, between saponins and antioxidant enzyme SOD, and between tannins and APX. The lowest positive correlation was 0.6 between TFC and the CAT antioxidant enzyme. In addition, a strong negative correlation of −1.00 was observed between alkaloids and the antioxidant enzyme APX and between alkaloids and tannins, while a weak negative correlation of −0.79 was observed between the alkaloids and TPC.

### 3.3. Molecular Analysis

Molecular markers were used for estimating the genetic diversity among the studied accessions of *C. gileadensis* using ten ISSR primers and eleven SCoT primers. For the ISSR marker, ten primers were used with a molecular size ranging from 192 to 1190 bp. The DNA profile generated from the ISSRs is shown in Figure S1. The ISSR primers produced 70 total number of bands. ISSR 5 produced the highest total number of bands and resolving power (RP) with values of 11 and 14.67, respectively, while ISSR 7 produced the lowest total number of bands and RP with values of 3 and 3.33, respectively. ISSR 10 showed the highest number of unique bands (6). Furthermore, primer ISSR 10 had the highest value for polymorphism information content (PIC), effective multiplex ratio (EMR), marker index (MI) and polymorphism percentage (P%) with values of 0.72, 7.11, 5.12 and 88.89%, respectively. Primer ISSR 3 had the lowest values for PIC, EMR, MI and P% with values of 0.08, 0.14, 0.01 and 14.29%, respectively, as presented in Table 2. 

Data for the SCoT marker are presented in Table 2; the DNA profile produced from the 11 primers is illustrated in Figure S2. The SCoT primers generated a total of 138 bands with a molecular size that varied from 63 to 1950 bp. Primer SCoT 5 showed the highest total number of bands with 20 bands, and the highest RP of 25.33. Primer SCoT 4 showed the lowest total number of bands with five bands and the lowest RP was eight. Primer SCoT 7 showed the highest result for PIC, EMR, MI and P (%) with values of 0.77, 19.00, 14.63 and 100%, respectively. Primer SCoT 1 showed lowest result for PIC, EMR, MI and P (%) with values of 0.19, 1.00, 0.19 and 33.33%, respectively (Table 2).

The molecular SCoT marker revealed a higher polymorphism percentage than ISSR with values of 75.36% and 55.71%, respectively (Table 3). Cluster UPGAMA using molecular data from the ISSR and SCoT markers showed the studied accessions classified into two groups; one group contained accessions C1 and C2 and other group contained C3 accession (Figure S3).

### 3.4. SDS-PAGE Protein Profile

The protein profiles of the studied accessions using SDS-PAGE are shown in Figure S4. Analysis of the SDS-PAGE generated a total of seven bands with molecular weight varying from 56 to 137 Kda. Two polymorphic bands were detected with two unique bands for accession C1 collected from Jeddah. Four bands appeared in C2 collected from Jizan and C3 collected from Riyadh showed no polymorphic bands. The protein polymorphism (PP%) between the studied accessions was 28.5% as shown in Table 3.

### 3.5. Relative Gene Expressions

**Figure 6:**
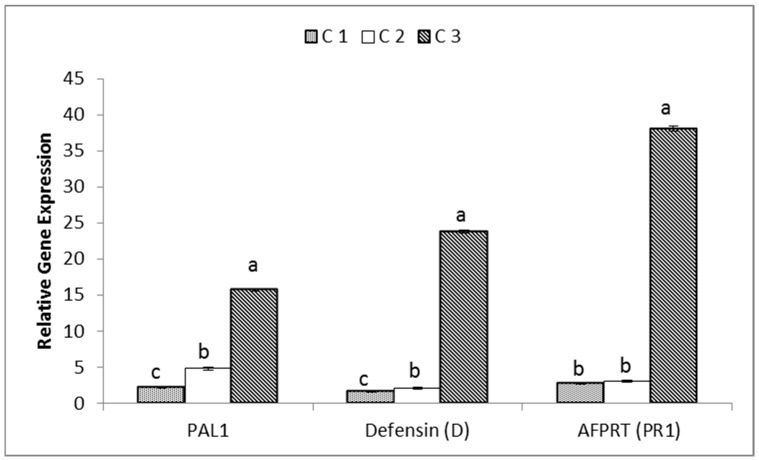
Relative gene expressions for PAL1, defensing (PR-12) and AFPRT (PR-1) genes in *C. gileadensis* accessions in KSA. Bars with different letters indicate significant differences between accessions, expressed as the mean of three replicates ± SDs.

The relative gene expressions for phenylalanine ammonia-lyase (PAL1), defensin (D) and pathogenesis-related protein (AFPRT) genes for the studied accessions collected from different locations were studied to estimate the effect of environmental conditions and different habitats in the regulation of genes that might assist genetic characterization and identifying variability. Gene expression data is presented in Figure 6, and shows that the PAL1, defensin and AFPRT genes were upregulated in accession C3, collected from Riyadh, with values of 15.68, 23.8 and 38.12, respectively, whereas in accession C1, collected from Jeddah, the genes were downregulated, showing values of 2.16, 1.63 and 2.76 for PAL1, D and AFPRT, respectively.

### 3.6. Data Analysis 

**Figure 7:**
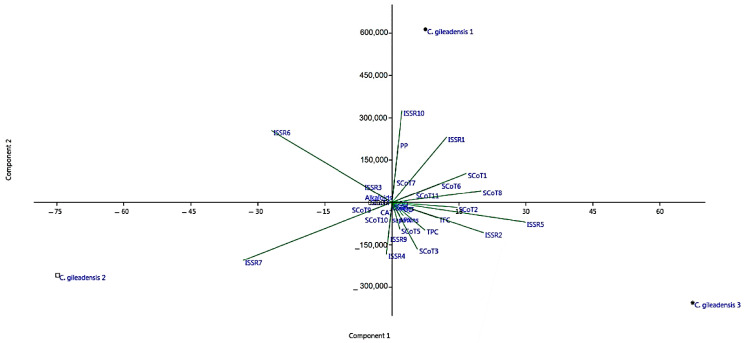
Principle component analysis (PCA) for all data attributes (phyto-biochenical parameters and molecular characteristics) for *C. gileadensis* accessions in KSA.

A principal component analysis (PCA) for phyto-biochemical, antioxidant enzyme activity parameters and molecular data was conducted, as shown in Figure 7. The two principal axes, Pca and PC2, had a total variance of 64.09% and 35.92%, respectively, with an eigenvalue of 1.34. Eigenvalues are used to measure the ordination quality and strength of phyto-biochemical, antioxidant enzyme parameters and molecular data relationships within the studied accessions. Data from the PCA revealed that the ISSR markers (ISSR 1, 2, 5, 6, 7 and 10) and SCoT markers (SCoT 1, 2, 3 and 8) were the most significant parameters followed by TPC, TFC and PP%. The arrow length indicates that the molecular attributes are the most powerful parameters, whereas the direction of the arrow suggests that the phyto-biochemical parameters were the most powerful ordinations. 

**Figure 8:**
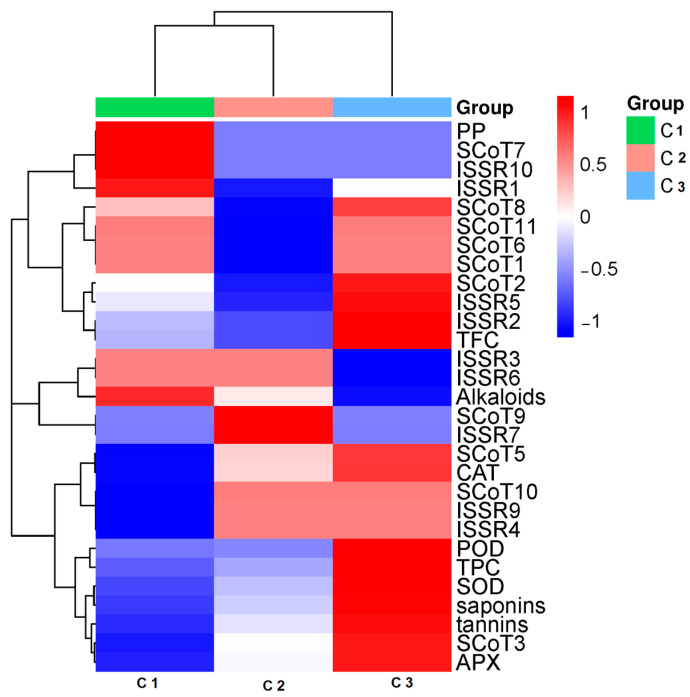
Heatmap displaying the relationship between *C. gileadensis* accessions in KSA and their phylogenetic relationships in relation to phyto-biochemical parameters and molecular markers. The studied taxa are indicated by the upper bar in three colors. The color scale denotes the variable level increasing from 1 (red) to −1 (blue).

Results from the heatmap cluster analysis showed the variations of phyto-biochemical, antioxidant enzyme activity and molecular attributes for the studied accessions from different locations (Figure 8). The studied accessions, divided horizontally into two clusters, had accessions C1 and C2 in first cluster and accession C3 in the second cluster, whereas the phyto-biochemical parameters and molecular data were divided vertically into two three clusters. The first cluster was divided into two sub-clusters; the first sub-cluster included phyto-biochemical and antioxidant enzyme activity parameters, whereas the other two clusters included the molecular attributes (ISSR and SCoT) and protein polymorphisms (PP%). 

## 4. Discussion

A DNA barcode should have a “barcode gap” between intraspecific and interspecific divergences, be routinely retrievable with a single primer pair with no need for manual editing of sequence traces and be able to offer the greatest amount of species differentiation [47,48,49]. The morphological and phytochemical traits of plants can be quickly changed by their geographic environment. Taxonomic identification of plants based on morphol-phytochemical characteristics necessitates a significant level of experience and expertise in plant taxonomy [50]. DNA based species-specific molecular genotyping (DNA barcoding) is a successful alternative to traditional phyto-morphological methods for successfully identifying species across all groups of live forms because DNA sequences are not affected by the environment and remain constant even during developmental stages [51]. In this study, phylogenetic analysis using ITS and 18 S rRNA regions showed that both nuclear regions were useful for the identification and estimation of molecular variations among the studied accessions from different natural habitats with highest pairwise identity 100%. These data were in agreement with the findings of Ali [52], who studied DNA barcoding for the *Commiphora* species in KSA using the nuclear ITS gene. Due to their highly conserved flanking regions, rRNA gene sequences are typically simple to access and employ with universal primers. Even in the tiniest species, their repeating structure inside the genome provides an abundance of template DNA for PCR. In plants, the 18 S gene, which is a component of the ribosomal functional core, is subject to similar selective pressure [53].

Plant scientists have been interested in phytochemical research as a result of the advancement of cutting-edge methodologies. These methods are crucial in the effort to find new sources of raw materials for the pharmaceutical sector. Phytochemical parameters are used in plant taxonomy and in studying diversity among species from same genus or in the same species from different localities [54]. In this investigation, TPC, TFC, tannins and saponins held the highest value for accession C3 collected from Riyadh. This result was consistent with the findings of Al-mahbashi [55] who studied the phytochemical composition and biological activity of *C. gileadensis* in Yemen. 

The detoxification of ROS in plants involves both enzymatic and non-enzymatic mechanisms. ROS can oxidatively damage various cellular components, such as membrane lipids, proteins, and nucleic acids [56]. This detoxification of ROS in plants involves numerous antioxidant compounds and enzymes. In this study, a large variation in the antioxidant enzyme activity of *C. gileadensis* accessions was discovered. With regard to habitat type and salt sensitivity, the enzyme activities (SOD, POX, CAT, and APX) were shown to be constitutively greater in the Ryiadh salt-tolerant habitat. The scavenging of H_2_O_2_ by CAT, POD, GPX and APX has been observed in several plant species [57]. The main enzymatic H_2_O_2_ scavenger in plants under salt stress, when the cellular H_2_O_2_ level increases many times greater than that of plants cultivated under normal conditions, may be CAT, which has a lower affinity for H_2_O_2_ but a higher processing rate than APX, GPX or POD [58]. The enzyme activity of CAT, POD, APX and GPX is significantly down regulated in salinity stressed plants [59].

The SDS-PAGE method is particularly trustworthy because the storage proteins are independent of environmental ups and downs, making protein electrophoresis a powerful and frequently used tool for population genetics. A precise genetic diversity measure is assessed using biochemical markers [26,60,61]. Data from the SDS-PAGE in this study revealed the accessions had the lowest polymorphisms and classified them into two groups. 

In this study, the effectiveness of ISSR and SCoT markers for analyzing genetic diversity and establishing genetic relationships between *C. gileadensis* accessions was observed by producing the highest polymorphism percentage by SCoT marker. Although multilocus DNA markers (such ISSRs and SCoTs) are frequently used across a wide range of plant species and genera, the effectiveness of their application can vary greatly depending on the type of plant used [62,63]. The molecular marker ISSR revealed 55.71% polymorphism with a total of 70 bands, whereas ISSR 8 provided a more efficient result with 100% polymorphism. The polymorphism percentage produced from the ISSR marker was lower than the polymorphism percentage produced from the ISSR marker used for genetic diversity for *C. wightii* in India, generated from 16 ISSR primers [64]. According to Geetha et al. [65] and Kawane et al. [66], plant reproductive behavior can be attributed to the limited genetic variability found within the analyzed *C. gileadensis* accessions. 

On the other hand, the SCoT markers make differences much more precise in a given germplasm collection by revealing the genetic diversity at the gene level, therefore boosting the likelihood of discovering new alleles [67]. Its capacity to identify polymorphism among members of a population is known as polymorphism information content (PIC). The output from the calculations gives us a PIC maximal value of 0.5 for dominant markers. However, several studies have reported a PIC value for dominant markers higher than 0.5 [28,68,69,70]. The value of PIC from the ISSR marker was 0.41, whereas the value from the SCoT marker was 0.52. Furthermore, the data from the SCoT marker was more informative compared with the data reported by Botstein et al. [71] which was moderately informative. In this study, MI and PIC data generated from the SCoT marker was higher than the MI and PIC generated from the ISSR marker. This result was in agreement with the result of PIC generated from the ISSR marker, which was higher than the PIC produced from the SCoT marker [72,73]. 

According to population theory, genes with high diversity should have balanced polymorphisms that signify adaptive variation. Gene expressions, with regard to isolation of numerous genes, whose expression did not differ between the plants in different natural habitats, according to real-time PCR, demonstrate that it is a perfect tool for enriching differentially expressed and novel genes. This is despite the fact that some of these genes’ expressions or functions were not clearly differentiated. In this study, three genes PAL1, defensin (D) and AFPRT were used to study diversity among *C. gileadensis* from three different habitats. Accession C3, collected from Riyadh, showed the expression of all genes were upregulated and folded two-fold more than other two accessions. The production of secondary metabolites by phenylalanine ammonia-lyase (PAL) genes, which regulate the plant growth response, is crucial for plant growth, development, adaptation and mitigation responses to various environmental and pathogenic stresses [74,75]. The study of the stress-inducible regulation of the defensin (PR-12) gene has identified many important conserved cis-elements, responsive to biotic and abiotic stresses [76]. In addition to AFPRT protective functions, they play a role in cell growth and plant hormone regulation. After reviewing aspects of this PR study, it was observed that the expression of PR1 genes has been extensively studied in response to different types of stresses and habitat compositions in various plant species [77].

## 5. Conclusions

The present study showed DNA nuclear regions (ITS and 18 S rRNA) have efficient power for use as a tool for DNA barcoding for identification of *C. gileadensis* accessions. Genetic diversity between the studied accessions identified that the C3 accession showed the highest value from the phytochemical parameters (TFC, TPC, saponins and tannins). Additionally, the highest data revealed from enzyme activity of SOD, POD, CAT and APX were present in the C3 accession from Riyadh. The molecular marker SCoT was more efficient, and it showed the highest polymorphism percentage compared to the ISSR marker; the gene expressions of PAL1, defensin, (PR12) and AFPRT (PR1) genes were upregulated in accession C3 depending on the environment tolerance. These findings are crucial for characterizing the germplasm and hunting for markers linked to critical *C. gileadensis* features for trait-oriented breeding.

## Figures and Tables

**Table 1:** Data generated from nuclear DNA region (ITS and 18 S) for the studied *C. gileadensis* accessions.

Nuclear Region	Accessions	GC%	GenBank Accession No.	Pairwise Identity (PI)	Nucleotide (bp)
ITS	C1	56%	OL411968	100%	1517
	C2	62.9%	ON129562	100%	420
	C3	64.3%	ON128707	95.45%	686
18 S	C1	50.1%	ON171463	100%	827
	C2	62.7%	ON176685	91.13%	859
	C3	50.9%	ON148092	100%	753

**Table 2:** Data generated from ISSR and SCoT markers for *C. gileadensis* accessions in KSA.

Marker	Primer Name	Sequence	Fragment Size (bp)	MB	PB		TB	PIC	EMR	MI	RP	P%
					UB	NB						
ISSR	ISSR1	(AG)8T	192–905	4	4	1	9	0.46	2.78	1.28	12.00	55.56
	ISSR2	(GA)8T	210–895	5	3	1	9	0.36	1.78	0.64	13.33	44.44
	ISSR3	(CT)8T	196–720	6	0	1	7	0.08	0.14	0.01	13.33	14.29
	ISSR4	(CT)8A	326–515	3	0	1	4	0.14	0.25	0.04	7.33	25.00
	ISSR5	(CA)8T	245–995	4	4	3	11	0.47	4.45	2.09	14.67	63.64
	ISSR6	(GT)8A	200–420	2	0	3	5	0.33	1.80	0.59	8.00	60.00
	ISSR7	(AG)8C	365–475	1	2	0	3	0.59	1.33	0.79	3.33	66.67
	ISSR8	(AG)8G	242–645	3	2	2	7	0.41	2.29	0.94	10.00	57.14
	ISSR9	(GA)8C	364–764	2	3	1	6	0.54	2.67	1.44	7.33	66.67
	ISSR10	(CT)8G	294–1190	1	6	2	9	0.72	7.11	5.12	8.67	88.89
Total				31	24	15	70	4.10	24.60	12.94	97.99	55.71
Average				3.10	2.40	1.50	7	0.41	2.46	1.29	9.80	55.71
SCoT	SCoT1	CAACAATGGCTACCACC	180–740	6	0	3	9	0.19	1.00	0.19	0.19	33.33
	SCoT2	CAACAATGGCTACCACG	200–785	2	11	0	13	0.75	9.31	6.98	0.75	84.62
	SCoT3	AAGCAATGGCTACCACC	203–385	3	2	2	7	0.41	2.29	0.94	0.41	57.14
	SCoT4	ACGACATGGCGACCAAC	195–428	3	1	1	5	0.29	0.80	0.23	0.29	40.00
	SCoT5	ACCATGGCTACCACCGA	63–925	5	7	8	20	0.53	11.25	5.96	0.53	75.00
	SCoT6	CACCATGGCTACCACCA	186–1430	1	8	5	14	0.71	12.07	8.57	0.71	92.86
	SCoT7	ACCATGGCTACCACCGC	189–1950	0	12	7	19	0.77	19.00	14.63	0.77	100.00
	SCoT8	ACGACATGGCGACCCAC	80–1040	3	10	4	17	0.66	11.53	7.61	0.66	82.35
	SCoT9	CCATGGCTACCACCGCA	185–1011	4	3	5	12	0.45	5.33	2.40	0.45	66.67
	SCoT10	ACGACATGGCGACCGCG	192–1018	5	2	5	12	0.41	4.08	1.67	0.41	58.33
	SCoT11	CAACAATGGCTACCACCC	260–1050	2	3	5	10	0.54	6.40	3.46	0.54	80.00
Total				34	59	45	138	5.71	83.06	52.64	166.66	75.36
Average				5.36	4.09	12.55	5.36	0.52	7.55	4.79	15.15	75.36

MB: monomorphic bands, UB: unique bands, NB: non-unique bands; PB: Polymorphic bands, TB: total bands, PIC: polymorphism information content, EMR: effective multiplex ratio, MI: marker index, RP: resolving power, and P (%): polymorphism percentage.

**Table 3:** Number of primers, size range, total bands and polymorphic bands produced by ISSR and SCoT markers used in this study.

Features	Molecular Markers		SDS-PAGE Protein
	ISSR	SCoT	
Band size range	192–1190 bp	63–1950 bp	56–137 KDa
Total bands	70	138	7
Polymorphic bands	39	104	2
Unique bands	8 (C1), 4(C2), 12 (C3)	23 (C1), 7(C2), 29 (C3)	2 (C1), 0 (C2 & C3)
% Polymorphism	55.71%	75.36%	28.5%

## Data Availability

Relevant data applicable to this research are within the paper.

## Supplementary Materials

The following supporting information can be downloaded at: https://www.mdpi.com/article/10.3390/genes13112099/s1, Figure S1: DNA profiles generated from ISSR marker for the *C. gileadensis* accessions in KSA; Figure S2: DNA profiles generated from SCoT marker for the *C. gileadensis* accessions in KSA; Figure S3: UPGAMA cluster for *C. gileadensis* accessions in KSA based on molecular markers (ISSR &SCoT); Figure S4: Protein profile of SDS-PAGE for *C. gileadensis* accessions in KSA; Table S1: Code and locations of *C. gileadensis* in KSA; Table S2: Primer sequence used in real time PCR for gene expression.
