# Supplementary material for: Genetic Characterization of Some Saudi Arabia’s Accessions from Commiphora gileadensis Using Physio-Biochemical Parameters, Molecular Markers, DNA Barcoding Analysis and Relative Gene Expression
Source: Genes (Basel). 2022 Nov 11;13(11):2099. doi: 10.3390/genes13112099 (PMC9690626; doi:10.3390/genes13112099)
Supplement: Supplementary file 1 [file genes-13-02099-s001.zip › genes-1994238-supplementary.pdf]

Genetic Characterization of Some Saudi Arabia's Accessions from *Commiphora gileadensis* Using Physio-Biochemical Parameters, Molecular Markers, DNA Barcoding Analysis and Relative Gene Expression

Fatmah Ahmed Safhi [1], Salha Mesfer ALshamrani [2], Areej Saud Jalal [1,*], Diaa Abd El-Moneim [3], Amal A. Alyamani [4] and Amira A. Ibrahim [5]

[1] Department of Biology, College of Science, Princess Nourah bint Abdulrahman University, P.O. Box 84428, Riyadh 11671, Saudi Arabia
[2] Department of Biology, College of Science, University of Jeddah, Jeddah 21959, Saudi Arabia
[3] Department of Plant Production, (Genetic Branch), Faculty of Environmental and Agricultural Sciences, Arish University, El-Arish 45511, Egypt
[4] Department of Biotechnology, Faculty of Science, Taif University, Taif 21974, Saudi Arabia
[5] Botany and Microbiology Department, Faculty of Science, Arish University, El-Arish 45511, Egypt
* Correspondence: Asjalal@pnu.edu.sa

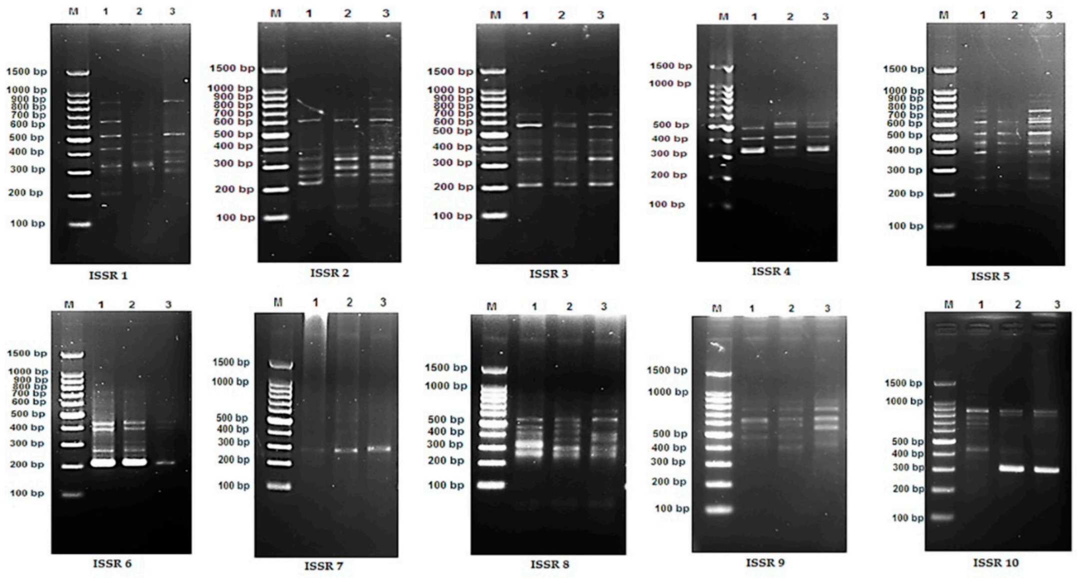

Figure S1. DNA profiles generated from ISSR marker for the *C. gileadensis* accessions in KSA.

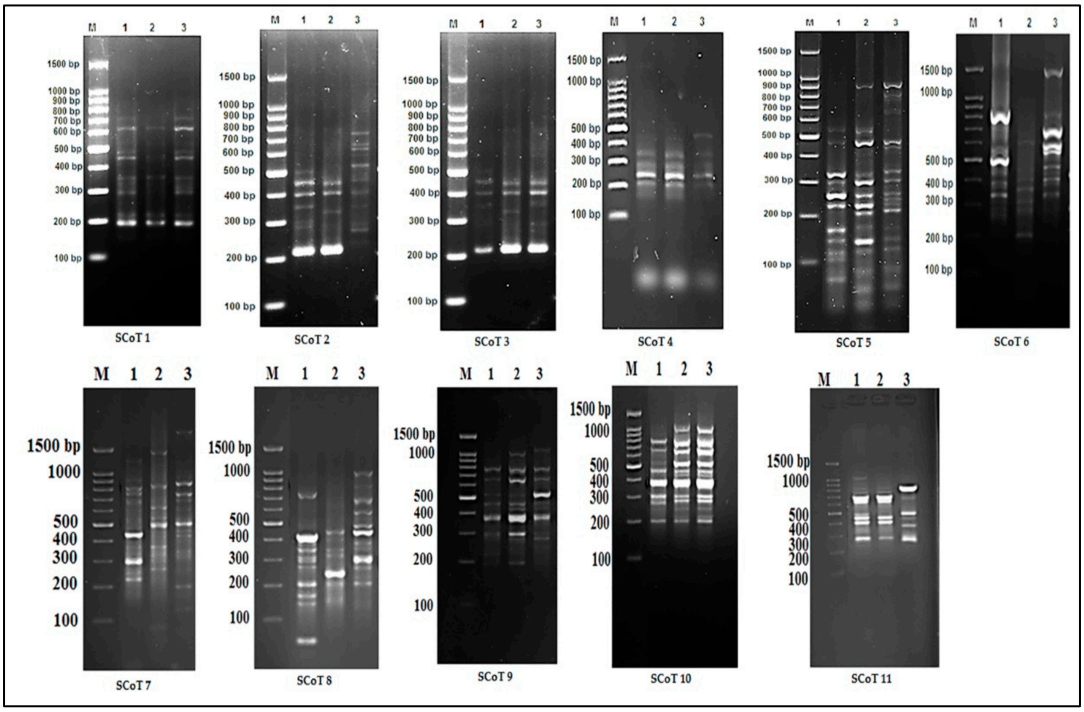

Figure S2. DNA profiles generated from SCoT marker for the *C. gileadensis* accessions in KSA.

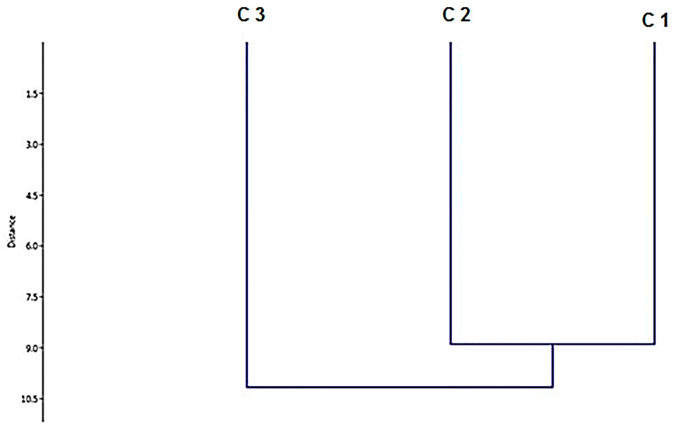

Figure S3. UPGAMA cluster for *C. gileadensis* accessions in KSA based on molecular markers (ISSR &SCoT).

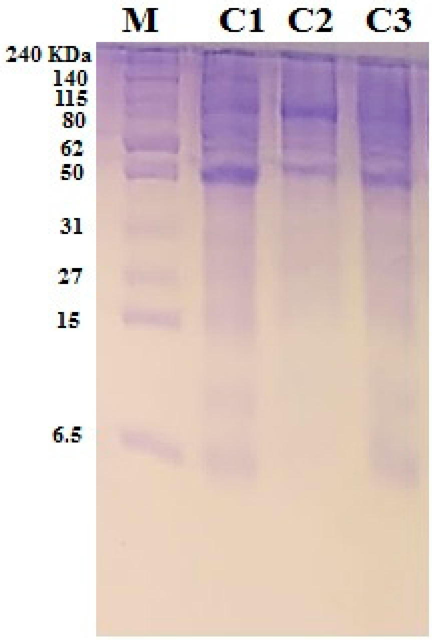

Figure S4. Protein profile of SDS-PAGE for *C. gileadensis* accessions in KSA.

Table S1. Code and locations of *Commiphora gileadensis* in KSA.

Code	Taxa	Location	Habitat	GPS Latitude (N)	Longitude €
C1	*Commiphora gileadensis*	Jeddah	High Salt tolerant	21° 29' 33.0000''	39° 10' 39.2520''
C2	*Commiphora gileadensis*	Jizan	Low ssalt tolerant	16° 53' 21.6924''	42° 34' 14.0412''
C3	Al-Baha *Commiphora gileadensis*	Riyadh	Low salt tolerant	24° 42' 48.7872''	46° 40' 31.0656''

Table S2. Primer sequence used in real time PCR for gene expression.

Primer		Sequence
PAL 1	F	5′-ACAAATGGACATRTTAAT-3′
	R	5′-CTTCTATGAGATGTTGC-3′
Defensin (PR-12)	F	5'-CCAAATGCCTCGTCATCT-3'
	R	5'-ATTAGAGTCAAGCTCAAAAGG-3'
AFPRT (PR1)	F	5'-ATGGAACACGACACTGGCAG-3'
	R	5'-GCATACTGACCAGAGTAACTGG-3'
β-Actin	F	5′-GTGCCCATTTACGAAGGATA- 3
	R	5′-GAAGACTCCATGCCGATCAT- 3′
